# Levels of physical activity before and after stroke in relation to early cognitive function

**DOI:** 10.1038/s41598-021-88606-9

**Published:** 2021-04-27

**Authors:** Adam Viktorisson, Elisabeth M. Andersson, Erik Lundström, Katharina S. Sunnerhagen

**Affiliations:** 1 Institute of Neuroscience and Physiology, Rehabilitation Medicine, Sahlgrenska Academy, University of Gothenburg, and the Sahlgrenska University Hospital, Per Dubbsgatan 14, 3rd Floor, PO Box 430, 405 30 Gothenburg, Sweden; 2 Department of Neuroscience, Neurology, Uppsala University, Uppsala, Sweden

**Keywords:** Neuroscience, Cognitive neuroscience

## Abstract

Regular physical activity is widely recommended in the primary and secondary prevention of stroke. Physical activity may enhance cognitive performance after stroke, but cognitive impairments could also hinder a person to take part in physical activity. However, a majority of previous studies have not found any association between post-stroke cognitive impairments and a person’s subsequent level of activity. In this explorative, longitudinal study, we describe the intraindividual change in physical activity from before to 6 months after stroke, in relation to early screening of post-stroke cognitive impairments. Participants were recruited at 2 to 15 days after stroke, and screened for cognitive impairments using the Montreal Cognitive Assessment tool. Information on pre-stroke physical activity was retrospectively collected at hospital admittance by physiotherapists. Post-stroke physical activity was evaluated after 6 months. Of 49 participants included, 44 were followed up. The level of physical activity changed in more than half of all participants after stroke. Participants who were physically active 6 months after stroke presented with significantly less cognitive impairments. These results highlight that many stroke survivors experience a change in their physical activity level following stroke, and that unimpaired cognition may be important for a stroke survivors’ ability to be physically active.

## Introduction

Stroke is a major cause of death and disability in the world. Surviving a stroke entails new and challenging circumstances with a potentially large impact on quality of life and participation^1^. Many survivors experience some stroke-related sequelae, including loss of motor function, sensory deficits, cognitive impairments and psychological problems. About one in five also suffer a reoccurring stroke^2^. Consequently, improving the quality of life among stroke survivors, while preventing reoccurring cerebrovascular disease poses a big challenge today.

Regular physical activity decreases the risk of stroke, and is associated with less severe symptoms and better cognitive function in those affected^3–5^. In stroke-rehabilitation, physical activity and exercise are important components that improves cardiovascular health, gait, and upper-limb function^6–8^. Regular of physical activity is also likely to reduce the risk of stroke reoccurrence, and recommended as part of secondary stroke prevention by the American Heart Association^9^. Despite this, stroke survivors are less active and spend more time sedentary compared to stroke-free people of the same age^10,11^. Factors such as age, sex, pre-stroke physical activity, functioning, depression, fatigue, self-efficacy, and quality of life have previously been associated with the level of post-stroke physical activity^12,13^.

Post-stroke physical activity can improve cognitive performance among stroke survivors^14,15^. However, cognitive deficits, and even mild such, may hinder stroke survivors to fully understand and implement the recommendations of physical activity. A recent study found that better cognitive performance measured during the first months after stroke was associated with higher physical activity levels at a two-years follow-up^16^. However, several other preceding studies have not been able find associations between post-stroke cognitive function and physical activity^17–21^. In this study, we aim to describe how physical activity levels change from before to 6 months after stroke, in relation to post-stroke cognition.

## Methods

In this explorative and prospective, longitudinal study we collected data on all patients treated for stroke at the Sahlgrenska University Hospital in Gothenburg, who were also included in the Swedish Efficacy oF Fluoxetine—a randomisEd Controlled Trial in Stroke (EFFECTS; NCT02683213)^22^. The EFFECTS was an investigator-led, multicenter, randomized, placebo-controlled, double-blind, parallel group trial with the objective to investigate whether fluoxetine could improve recovery and quality of life after stroke. The data collection has been described previously^23^. In brief, adult stroke patients were prospectively identified and enrolled in the EFFECTS at 2–15 days after stroke onset. Each participant was randomly assigned to fluoxetine or placebo, which did not affect their functional outcome^24^. Randomization was conducted in a secure web-based system, using a minimization program to achieve balance between treatment groups. A follow-up was conducted at 6 months. Additional data were collected from the opt-out local Swedish Väststroke register at baseline and at a 3 months follow-up after stroke, and from inpatient medical records.

Inclusion of participants took place between October 20th 2014 and June 28th 2019. Inclusion criteria were age of 18 years or older, persistent focal neurological deficits, and a clinical stroke diagnosis with brain imaging supporting an intracerebral hemorrhage or ischemic stroke. Exclusion criteria were severe comorbidity or short life expectancy, contraindications to treatment with fluoxetine, and a current or recent diagnosis of depression. Patients who could not consent to participate for themselves, or unable to communicate in Swedish were also excluded.

### Setting

The comprehensive stroke units at Sahlgrenska University Hospital has a catchment area of 700,000 people, and patients who fulfill the criteria for thrombolysis or thrombectomy in nearby regions are transported to this hospital. All stroke patients are to be treated at the specialized stroke units with multi-professional teams consisting of nurses, physiotherapists, occupational therapists, speech and language therapists, psychologists, and social workers. In accordance with Swedish guidelines, all patients receive a minimum of 30 min training with a physiotherapist per day during the hospital stay. Individualized rehabilitation is planned and recommended for all patients prior to discharge.

### Physical activity assessments

The average level of physical activity during the year before the stroke was assessed at hospital admittance by physiotherapists working at the stroke units. Assessments were performed according to the Saltin-Grimby Physical Activity Level Scale (SGPALS), and recorded in the Väststroke register. The following question was used: “How much did you move or exercise during leisure time before stroke? Try to estimate an average”. In addition, the physiotherapists asked follow-up questions about intensity, duration and type of activity^5^. If possible, the information was confirmed by relatives or care-givers, using the same questions. The SGPALS has 4 levels: level 1, mostly sedentary (inactive); level 2, physical activity for at least 4 h weekly (light physical activity); level 3, regular physical activity and training for at least 2 h weekly (high physical activity); level 4, regular hard physical training for competition sports several times per week (very high physical activity)^25^. Physical activity levels were re-evaluated and categorized according to SGPALS at the 6 months follow-up. SGPALS level 4 was not reported by any participant, and thereby removed from figures and tables.

### Outcome variables

Cognitive function early after stroke was evaluated using Montreal Cognitive Assessment (MoCA)^26^. MoCA is a screening tool for detecting cognitive impairment, with a maximum score of 30 points. Lower scores indicate a more severe cognitive impairment. A cut-off score of > 25 in MoCA was applied for unimpaired cognitive function. Stroke severity was evaluated using National Institutes of Health Stroke Scale (NIHSS)^27^. NIHSS is a 11-item impairment scale with a maximum of 42 points. Higher scores indicate greater stroke severity. Assessments of MoCa and NIHSS were performed by a research nurse working at the stroke unit, at 2 to 15 days after stroke onset. Ambulation status was recorded using the Functional Ambulation Categories (FAC), based on evaluations performed by physiotherapists at hospital discharge. The FAC has 6 levels: level 0, non-functional walking; level 1, dependent walking with a high level of manual contact; level 2, dependent walking with a low level of manual contact; level 3, dependent walking with no manual contact but supervision; level 4, independent walking on level surfaces only; level 5, independent walking on all surfaces^28^. Data on post-stroke disability was collected from the Väststroke register, recorded at a 3 months follow-up using the modified Rankin Scale (mRS)^29^. The mRS has 7 levels: level 0, no symptoms; level 1, no significant disability; level 2, slight disability; level 3, moderate disability; level 4, moderately severe disability; level 5, severe disability; level 6, death. The mRS is widely used to classify disability, validated for post-stroke populations^30^. Information on comorbidities present before the stroke, and post-stroke rehabilitation was collected from inpatient medical records.

### Statistics

The results are presented using descriptive data. Frequencies are reported as number of participants with percentages for categorical values and median with interquartile range (IQR) for ordinal values. Wilcoxon signed rank’s test was performed to evaluate the interindividual change in physical activity levels over time. Chi-square test was used to compare differences in MoCA between physically active (SGPALS level 2–3) and inactive (SGPALS level 1) participants before and after stroke. All p-values are two sided and interpreted at a significance level of 0.05. Statistical analyses were executed with IBM SPSS Statistics V.25.0.

### Ethics

This study and the study protocol were approved by a central medical ethics committee in Stockholm (reference 2013/1265-31/2) and by the Swedish Medical Agency (reference 5.1-2014-43006). All participants provided written informed consent. The Declaration of Helsinki was followed, and all participants were informed that they had the right to withdraw their participation at any time.

## Results

There were 49 stroke patients treated at the Sahlgrenska University Hospital, recruited in the EFFECTS study. Forty-four (90%) were followed up at 6 months. Among those lost to follow-up, two had died, one declined further participation and two were lost due to logistic errors. Characteristics of the participants were collected at baseline, and are presented in Table 1. The majority of participants had a mild stroke, and only six participants had a NIHSS score above 5 with individual scores of 6, 7, 8, 10, 17 and 18. There were six participants with a NIHSS score of 0. At hospital discharge, 19 participants were independent walkers, 23 were dependent walkers, and two were not able to walk. Almost all participants (n = 41) received rehabilitation after hospital discharge, and 28 had no or non-significant disability at 3 months post stroke.

**Table 1:** Characteristics for study population, devided by their physical activity level 6 months after stroke.

Characteristics	Participants	
	Physically inactive after stroke (n = 15)	Physically active after stroke (n = 29)
Female sex, n (%)	6 (40)	6 (21)
Age, median (IQR)	70 (65–76)	67 (59–73)
Smoking, n (%)	2 (13)	4 (14)
Ischemic stroke, n (%)	12 (80)	23 (79)
Haemorrhagic stroke, n (%)	3 (20)	6 (21)
NIHSS, median (IQR)	2 (1–5)	3 (1–4)
**Pre-stroke morbidity, n (%)**		
Stroke or TIA	4 (27)	3 (10)
Coronary heart disease	5 (33)	2 (7)*
Atrial fibrillation	3 (20)	4 (14)*
Hypertension	8 (53)	13 (45)
Diabetes mellitus	6 (40)	2 (7)
Chronic pulmonary disease	1 (7)	2 (7)
Any tumor	1 (7)	1 (3)
History of depression	1 (7)	2 (7)
**Pre-stroke SGPALS, n (%)**		
Inactive	3 (20)	7 (24)
Light physical activity	11 (73)	16 (55)
High physical activity	1 (7)	6 (21)
**Post-stroke MoCA, n (%)**		
Unimpaired cognition (> 25)	2 (13)	14 (48)
Impaired cognition (≤ 25)	13 (87)	15 (52)
**FAC at discharge, n (%)**		
Non-functional walking	1 (7)	1 (3)
Dependent walking	10 (67)	13 (45)
Independent walking	4 (27)	15 (52)
**mRS at 3 months, n (%)**		
No disability	1 (7)	5 (17)
Non-significant disability	7 (47)	15 (52)
Slight disability	5 (33)	6 (21)
Moderate disability	2 (13)	3 (10)
**Rehabilitation, n (%)**		
Highly specialized rehabilitation	6 (40)	15 (52)
Primary care rehabilitation	3 (20)	13 (45)
Geriatric rehabilitation ward	3 (20)	0 (0)
Rehabilitation at a nursing home	1 (7)	0 (0)
No rehabilitation	2 (13)	1 (3)
Days in hospital, median (IQR)	16 (10–41)	13 (7–30.5)
Discharged to own home, n (%)	8 (53)	20 (69)

*Variables with missing data on one participant. Percentages are reported as valid percent.

*n* numbers of participants, *IQR* interquartile range, *NIHSS* National Institutes of Health Stroke Scale^27^, *MoCA*, Montreal cognitive assessment scale^26^, *FAC* Functional ambulation categories^28^, *mRS* modified Rankin scale^29^, *SGPALS* Saltin-Grimby activity level scale. *SGPALS Level 1*, mostly sedentary (inactive), *SGPALS Level 2* physical activity for at least 4 h weekly (light physical activity), *SGPALS Level 3* regular physical activity and training for at least 2 h weekly (high physical activity)^25^, *TIA* transient ischemic attack.

**Figure 1:**
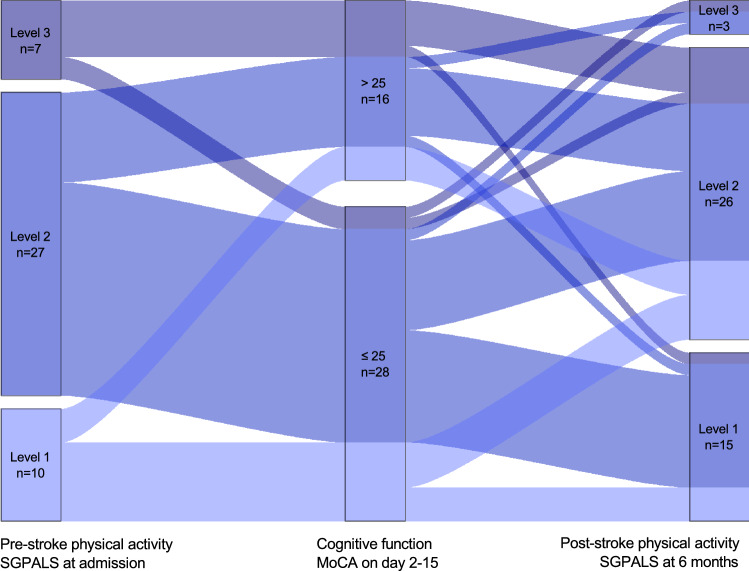
Path-diagram for the intraindividual change in physical activity level (SGPALS) relative cognitive function (MoCA). Abbreviations: MoCA; Montreal Cognitive Assessment Scale. SGPALS; Saltin-Grimby Activity Level Scale. MoCA score > 25: Unimpaired cognitive function. SGPALS Level 1: mostly sedentary (inactive); SGPALS Level 2: physical activity for at least 4 h weekly (light physical activity); SGPALS Level 3: regular physical activity and training for at least 2 h weekly (high physical activity).

The intraindividual change in physical activity, in relation to cognitive function can be seen in Fig. 1. Approximately one fourth of the study participants were physically inactive before their stroke, corresponding to one third 6 months after stroke. More than half (n = 26) of all participants had a shift in their activity level between the two time points. At the follow-up, nine were more physically active than before their stroke and 17 were less active. Among participants who were inactive before stroke, seven of 10 became more physically active after stroke. Of those registered as inactive at 6 months, 12 of 15 reported regular physical activity before stroke. Four of the 15 inactive participants were independent walkers at discharge, compared to 15 of the 29 physically active participants. No participant performed hard physical training (corresponding to SGPALS level 4), neither at baseline, nor at the follow-up. There was no statistically significant difference in physical activity levels before and after stroke in the study population (Z =  − 1.671, *p* = 0.095).

**Figure 2:**
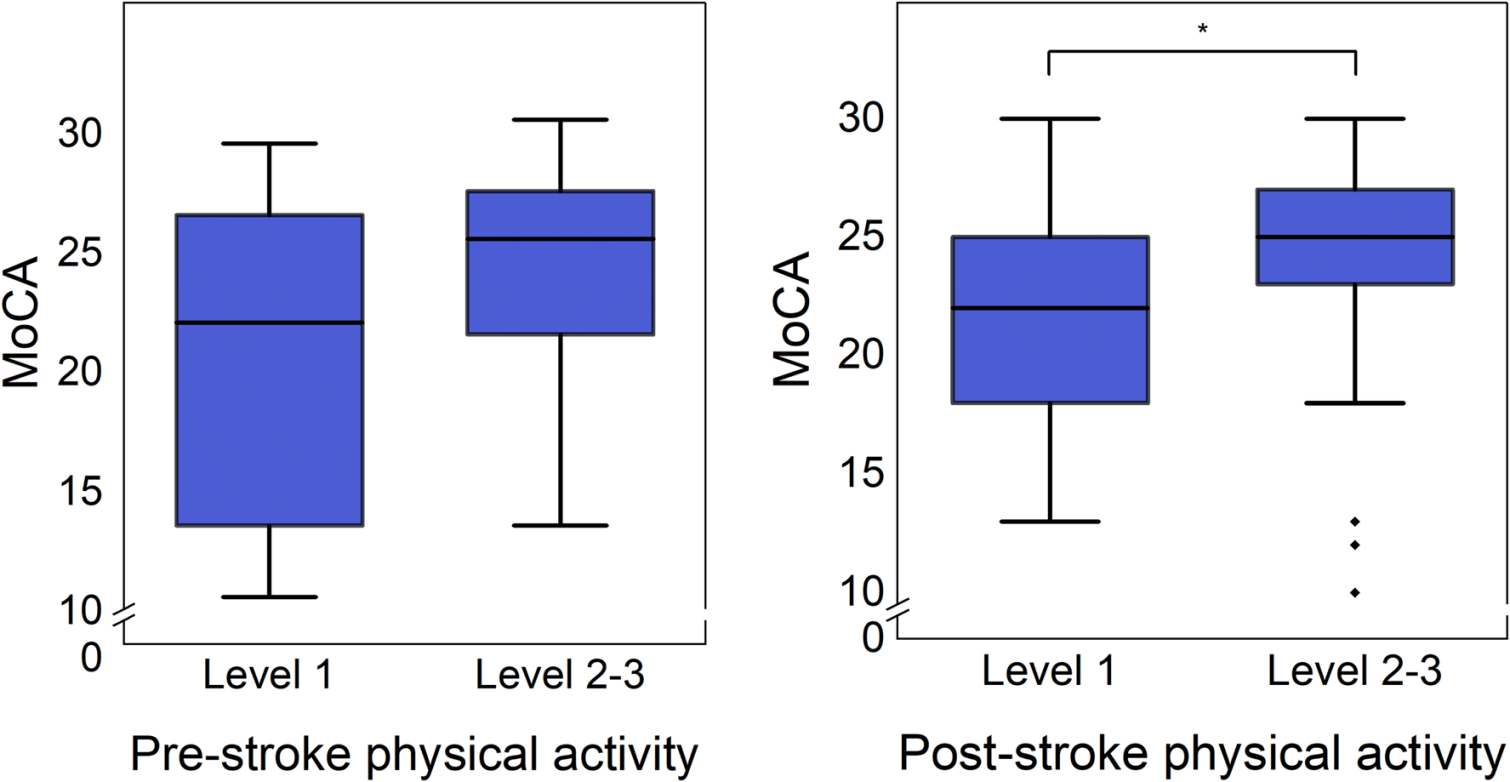
Box and whisker plots for comparisons in cognitive function (MoCA) between physically inactive (SGPALS level 1) and active (SGPALS level 2–3) participants before stroke, and six months after stroke. *Indicates statistical significance (*p* < 0.05). Abbreviations: MoCA; Montreal Cognitive Assessment Scale. SGPALS Level 1: mostly sedentary (inactive); SGPALS Level 2: physical activity for at least 4 h weekly (light physical activity); SGPALS Level 3: regular physical activity and training for at least 2 h weekly (high physical activity).

Early cognitive impairment was recorded for 10 of 12 participants that went from regular physical activity before stroke to being inactive after stroke. Among those physically activie after stroke, about half (14 of 29) had unimpaired cognitive function, compared to only two of 15 among those who were inactive after stroke. Interestingly, one patient suffering from a severe stroke with a MoCA score of 13 went from being inactive before stroke to regular physical activity after stroke. Comparisons in MoCA scores between physically active and inactive participants can be seen in Fig. 2. Stroke survivors that were physically active 6 months post-stroke presented with a median MoCA score of 25 (IQR: 22-27.5) compared to 22 (IQR: 18-25) among those who were physically inactive, and the difference was statistically significant (χ^2^ = 5.216, *p* = 0.045).

## Discussion

This study describes how the physical activity levels of 44 stroke survivors change from before, to 6 months after stroke. Half of all participants reported a change in their activity level following stroke. Notably is that the majority of participants with a sedentary lifestyle prior to stroke were physically activity at 6 months. Although the reason behind this remains unclear, it could be an effect of successful rehabilitation or an increased motivation to undertake health-beneficial life-style changes following stroke. Still, a large proportion of participants reported a decrease in physical activity as well. The substantial change in activity level after stroke is in line with the results of a prior, larger study^31^. Another recent study did, on the other-hand, report that all stroke survivors who were physically active before stroke remained active 6 months after stroke^19^. These conflicting results convey that there may be large differences between stroke populations and the health-care interventions they receive, but may also be a result of different assessment methods for physical activity.

There were a few more inactive participants at 6 months after stroke, compared to before stroke, but we found no significant difference between pre- and post-stroke physical activity on a group level. This result is in line with two previous studies^13,31^. Regular pre-stroke physical activity has been found to predict a better functional outcome after stroke^32,33^. Although this was not apparent in this study, several factors beyond functioning may, however, affect whether a stroke survivor will be physically active or not. Older age, depression, fatigue, insecurities, low quality of life and lack of motivation have been associated with an inactive lifestyle after stroke^12,20^. Among stroke survivors, difficulties with transportation and accessibility, embarrassment, health issues and fear of stroke reoccurrence may serve as self-perceived barriers for physical activity; whereas the possibility of support from others could motivate some to be physically active^34^.

An interesting finding in this study is that participants with early cognitive impairment were less active at 6 months. These results suggest that cognitive deficits may hinder the implementation of physical activity as secondary prevention. For clinicians, it may be important to identify and support these patients in order for them to reach an adequate level of physical activity. Conversely, previous studies investigating the relationship between post-stroke cognition and physical activity have reported inconsistent results, and the majority found no significant associations^16–21^. However, all studies on this topic, including this one, have been relatively small and lacking of sample size estimates. Hence the role of cognitive function for post-stroke physical activity needs further evaluation in future studies. We will hereafter investigate the relationship between early cognitive function and post-stroke physical activity in the complete EFFECTS cohort.

Longitudinal studies mapping physical activity levels before and after stroke are sparse. The strengths of this study are in its prospective and explorative design, with near-complete ascertainments and follow-up. Still, this study has several limitations. Firstly, the study population was drawn from a large intervention cohort, and conducted outside of the primary research objective. Due to the explorative nature of this study, no sample size calculation was performed on beforehand, and the statistical significance testing should therefore be interpreted with caution. The design of the original cohort also limits the generalizability of the study population, and thereby the applicability of the results. In addition, all participants were included from the same region in Sweden, and represent a small number of all stroke patients treated there during the study period. Most participants had a mild stroke, with low NIHSS scores, which also limits the generalizability. Another limitation is that information on physical activity was collected using a self-reported questionnaire. To this end, objective assessments of physical activity would have been more reliable, but difficult to obtain. The SGPALS questionnaire has a good concurrent validity, in comparison to objective measurements, and good predictive validity for various health-related risk factors, morbidity and mortality^35^. The SGPALS questionnaire is also widely used in clinical routine, and easy to use in order to find patients with inadequate physical activity. Lastly, the information on pre-stroke physical activity was collected retrospectively, which may introduce a recall bias. Moreover, participants with cognitive impairments may be less accurate in their indication of former physical activity, but this was at least partly handled by confirmatory questions to relatives and care-givers.

## Conclusions

A large proportion of stroke survivors experience a change in their physical activity level following stroke. Unimpaired cognition may facilitate a higher level of post-stroke physical activity, present 6 months after stroke. The importance of cognitive function for post-stroke physical activity requires further investigation, and should be evaluated in future studies.

## Data Availability

Data sharing is restricted due to the sensitive nature of the data. Researchers may apply for access to the data from Professor Katharina Stibrant Sunnerhagen (ks.sunnerhagen@neuro.gu.se). According to Swedish regulation: http://www.epn.se/en/start/regulations/, the permission to use data is only for what has been applied for and then approved by the Ethical board. To not follow the regulations is seen as scientific misconduct.
